# Strategies for incorporating indigenous placenta disposal methods in public healthcare: A Delphi study in Tshwane District, Gauteng, South Africa

**DOI:** 10.4102/hsag.v30i0.2988

**Published:** 2025-11-28

**Authors:** Cecilia Moeti, Fhumulani M. Mulaudzi, Molatelo M. Rasweswe

**Affiliations:** 1 Department of Nursing Science, Faculty of Health Sciences, Science, University of Pretoria, Pretoria, South Africa; 2 Department of Nursing Sciences, Faculty of Health Sciences, University of Limpopo, Polokwane, South Africa

**Keywords:** e-Delphi, expert, indigenous, placenta disposal, strategies

## Abstract

**Background:**

Indigenous placenta disposal strategies are essential to bridge clinical protocols with indigenous worldviews, allowing for safe and respectful handling without compromising health standards. Using Appreciative Inquiry in the Dream and Design phases, stakeholders collaboratively developed draft strategies that honour indigenous values while aligning with clinical protocols. These strategies further need inputs from a wider group of stakeholders to ensure that they fit both hospital rules and indigenous traditions.

**Aim:**

To refine and validate the draft strategies that incorporate indigenous placenta disposal methods for women birthing in the Tshwane District, Gauteng, South Africa.

**Setting:**

The Delphi process was conducted remotely with subject-matter experts based in Gauteng province, South Africa.

**Methods:**

Three iterative rounds of feedback using a modified e-Delphi technique were employed to ensure reliability. Twenty homogeneous experts were recruited to enrich validity. Of these, 14 participated in rounds one and two, while 10 participated in round three. For anonymity, questionnaires which included a 4-point Likert scale with five principles were sent through blind emails to the expert for them to rank the draft strategies. The consensus was set at 70% and the draft strategies that experts agreed on were repeated in the next rounds for further ranking until consensus was reached.

**Results:**

Five strategies that focused on fostering collaboration between midwives and indigenous women, infection prevention and control on indigenous placenta disposal, the development of culturally competent policies and guidelines in the healthcare facilities, the provision of culture competency training and awareness on cultural maternal care were refined and validated.

**Conclusion:**

These strategies may facilitate the safe indigenous placenta disposal while creating a harmonious space between midwives and indigenous women.

**Contribution:**

This study underscores the convergence of cultural values, healthcare policy, and sustainability, advancing culturally competent care by integrating Indigenous practices within contemporary medical frameworks.

## Introduction

The development of culturally sensitive strategies for indigenous placenta disposal within hospital settings emerged from long-standing tensions between biomedical protocols and indigenous birthing traditions. In many indigenous communities, the placenta holds deep spiritual and cultural significance, often requiring specific ceremonial handling and burial practices (Dickinson, Foss & Kroløkke 2017). However, mainstream hospital systems have historically lacked or limited frameworks to accommodate these practices, leading to distress, mistrust and cultural dissonance for indigenous families (Moeti, Mulaudzi & Rasweswe 2025). To address this gap, a participatory approach rooted in Appreciative Inquiry (AI) was employed. Through this process, in the Dream and Design phases of AI, a set of draft strategies was co-developed, reflecting both clinical feasibility and cultural integrity. These strategies further require validation through broader stakeholder engagement to ensure they are practical, respectful and aligned with both hospital policies and indigenous values.

Globally, the placenta holds a significant role in the post-delivery of a baby. It is seen as an organ that holds power, and its disposal should be handled with care and respect. The rituals surrounding the placenta disposal are common globally; however, they vary from culture to culture. In Korea, the placenta is often dried and hung or used to make traditional medicine, as they believe the placenta carries vitality and can be used as a wonder drug (Leepilyoung 2020). In the African continent, the Changa tribe in Tanzania believes that the hanging of the dried placenta signifies the continuity in the family (Eibel 2020). According to Rono, Maithrya and Sorre (2018), in Hungary, the placenta is burnt, and the ashes are placed in the men’s drinks with the belief that it makes the woman stop having children, which is similar to a study conducted among Korean women. In contrast to the latter, it was found that in Japanese culture, eating the placenta would increase the woman’s fertility (Rono et al. 2018).

In Western medicine, the placenta is treated as waste and discarded as such (World Health Organization [WHO] 2017). It also carries potential risks of infection if not handled appropriately. South Africa is known for its cultural diversity; hence, women who give birth in hospital settings should not feel marginalised by having their cultures undermined by midwives. According to the *Human Tissue Act*, women are allowed to take their placentas for indigenous disposal; however, midwives have limited or no guidelines to assist women in safe indigenous placenta disposal. This necessitates the incorporation of indigenous placenta disposal strategies into the healthcare system.

Incorporating indigenous placenta disposal practices into public healthcare systems, particularly for women birthing in the Tshwane District, Gauteng, requires a well-structured, contextually relevant approach. While South Africa’s *National Health Act* (No. 61 of 2003) and the *Traditional Health Practitioners Act* (No. 22 of 2007) support the integration of indigenous practices into formal health settings, the absence of specific, robust strategies for placenta disposal hinders meaningful progress. Without rigorously developed strategies, healthcare providers face challenges in respecting cultural practices while meeting clinical and public health standards. This gap highlights a need for structured, refined and validated strategies that bridge clinical protocols with the cultural expectations of indigenous communities. Globally, policies supporting culturally responsive healthcare are emerging, yet practical implementation remains inconsistent (Hudson, Hunter & Peckham 2019; Olcoń, Rambaldini-Gooding & Degeling 2023).

In sub-Saharan Africa, where indigenous beliefs strongly influence healthcare practices, the lack of integration of traditional customs within formal healthcare is especially pronounced. South Africa’s *Traditional Health Practitioners Act 22 of 2007* formally acknowledges the role of traditional healing, yet culturally significant practices, such as placenta disposal, are not explicitly addressed, leaving implementation ambiguous and inconsistent (Van Rooyen et al. 2017). Scholars argue that while policies recognise indigenous practices, Western medical practices still dominate, often sidelining indigenous customs in maternal and neonatal healthcare services (Musie et al. 2024).

The *National Health Act 61 of 2003*, Chapter 8, advocates for proper control of the use of blood, blood products, tissue and gametes in humans, yet their translation into practical healthcare delivery, particularly regarding practices like placenta disposal, remains limited. In the Tshwane District, Gauteng province, where many residents consider placenta disposal a sacred act, public healthcare facilities often lack formalised strategies to accommodate such practices, contributing to cultural dissonance within healthcare delivery (Moeti 2024).

This study employed the AI 5-D model: Define, Discover, Dream, Design, focusing on the final phase, Destiny. The AI model guided the development of culturally sensitive strategies, beginning with the Define phase, which clarified the need for indigenous placenta disposal practices within public healthcare. The Discovery phase facilitated the exploration of community expectations and ideal outcomes, grounding the strategies in both healthcare standards and indigenous cultural values. In this AI phase, where 10 Midwives and 10 Traditional Birth Attendants (TBAs) and/or Indigenous Knowledge Holders (IKHs) participated in a qualitative study, it was discovered that women in the Tshwane district do request their placenta for indigenous disposal at home and yet midwives had no guidelines to follow when releasing these placentas (Moeti et al. 2025).

Moreover, the AI Dream and Design phases were particularly influential, as 10 Midwives and 10 TBAs and/or IKHs collaboratively conceptualised strategies that balanced cultural relevance with clinical practicality. However, strategy creation alone does not guarantee success in complex, culturally sensitive healthcare environments. Strategies must undergo refinement and validation to ensure their adaptability to the specific healthcare framework (Driver 2014), allowing effective, culturally aligned care that meets the needs of both patients and providers. For this purpose, expert consensus is indispensable, as it brings together varied perspectives to produce strategies that are not only scientifically sound but also relevant and sensitive to the socio-cultural dynamics of the target community.

Finally, the Destiny phase enabled the transition from conceptual design to actionable, sustainable implementation, underscoring the importance of strategies that are both feasible within the healthcare system and deeply resonant with community practices. The study aims to refine and validate the draft strategies developed in the AI Dream and Design phases.

## Research methods and designs

Ogbeifun, Mbohwa and Pretorius (2016) indicated that the e-Delphi process, renowned for its effectiveness in achieving consensus on complex issues, allowed for anonymous, structured input from a diverse panel of stakeholders, ensuring that each round brought greater clarity, refinement and contextual adaptation. This study employed the e-Delphi method to tap into expert knowledge and build consensus on the subject studied. This method’s adaptability to culturally sensitive contexts makes it especially suited to the current study, as it allows for an inclusive, evidence-based approach to strategy validation (Ogbeifun et al. 2016).

### Study population and sampling

Participants were recruited through a variety of channels, that is, verbally, through email and telephonically. According to Bloor et al. (2015), the number of participants can range from as few as 4 to several thousand. Twenty experts who met the criteria were selected to gather informed insights and achieve consensus on a given topic (Taylor et al. 2016). The set criteria included experts with at least 5 years of relevant experience in maternal and neonatal healthcare practices and IKHs on the subject studied. This criterion was established to ensure that the selected experts possessed substantial knowledge and understanding of the cultural and medical implications surrounding placenta disposal. Importantly, the experts recruited for this phase did not participate in the initial qualitative study that drafted these strategies. This separation was intentional, allowing for an objective assessment of the strategies without bias from prior involvement.

The recruitment of experts employed a purposive sampling method, which allowed the researchers to select individuals based on their expertise and relevance to the topic and speciality rather than random selection. This approach was essential to ensure that the final recommendations would be grounded in a robust understanding of both indigenous practices and contemporary healthcare standards. By assembling a knowledgeable and diverse group of experts, this e-Delphi study aimed to create a comprehensive validation process that would enhance the cultural appropriateness and effectiveness of the strategies developed for placenta disposal in the Tshwane District. Table 1 outlines the characteristics of expert panellists.

**TABLE 1:** Characteristics of expert panellists.

No	Professional category	Highest qualifications	Experience in midwifery or indigenous births (years)
1	Registered nurse	Master of Nursing Science	15
2	Indigenous Knowledge Holder	Advanced Diploma in Basic Education	10
3	Advanced Midwife	Advanced Diploma in Midwifery	21
4	Midwife	Bachelor’s degree in nursing science	9
5	Community Health Worker	Diploma in Policing	7
6	Traditional Birth Attendant	Bachelor’s in Basic Education	12
7	Pharmacist assistant	Post Basic Diploma in Pharmacy	16
8	Traditional Birth Attendant	Certificate in Traditional Practice	18
9	Advanced Midwife	Advanced Diploma in Midwifery	11
10	Indigenous Knowledge Holder	Certificate in Nursing Assistant	22
11	Academia and Research	PhD in Nursing Science	17
12	Indigenous Knowledge Holder	Bachelor’s degree in social sciences	19
13	Academia and Research	PhD in Nursing Science	24
14	Medical Doctor	MBCHB	12

*Source*: Moeti, C., Mulaudzi, F.M. & Rasweswe, M.M., 2025, ‘Indigenous placenta disposal: The views of midwives and traditional birth attendants/indigenous knowledge holders in Tshwane District, Gauteng Province, South Africa’, *The Open Public Health Journal* 18, e18749445356679. http://dx.doi.org/10.2174/0118749445356679241122110424.

MBCHB, Bachelor of Medicine, Bachelor of Surgery.

### Data collection

In this study, data collection took place in three iterative survey rounds. Twenty experts who showed interest in participation were sent the survey information describing the objectives of the study and the survey validation instructions. Written consent was obtained from the expert participants before the commencement of the survey. Those who did not acknowledge receipt of the information were followed up with phone calls to confirm that they received the email. All communication was blinded to maintain anonymity and avoid bias.

### Survey rounds

The survey process involved three rounds of expert consultation to refine and validate five preliminary strategies. In the first round, the researcher emailed questionnaires to 20 experts using a 4-point Likert scale with a rating of 1 to 4 to rate the five principles. In this round, experts were asked to rate each strategy using the Likert scale and provide open-ended feedback, including suggestions for rephrasing or modifying the strategies. Fourteen responses were received after follow-ups. In the second round, revised strategies are presented based on feedback from round one. Experts re-evaluated these strategies, and the researchers compared the new ratings with the previous ones to identify shifts in opinion and movement toward consensus.

The second round involved sending the same questionnaires with controlled feedback to the 14 respondents to rate the five principles as in the first round, highlighting amended strategies and inviting further input; 10 experts responded. The third and final round involved presenting the updated strategies for final evaluation. The experts were asked to reconsider their judgements to reach consensus, with modified strategies highlighted for clarity. Again, 10 responses were received, and anonymity was maintained throughout the process.

### Data analysis

In a three-round Delphi technique using a 4-point Likert scale, where 1 indicates strongly agree and 4 indicates strongly disagree, data analysis involves both quantitative and qualitative methods to evaluate five key principles. The researchers used expert responses collected through the Likert scale to assess levels of agreement. These ratings were analysed quantitatively using statistical methods and percentage calculations to determine the overall consensus rate. Qualitative analysis was exercised by reviewing open-ended comments, where experts suggested rephrasing or modifications of some strategies. This was to identify common elements and areas for improvement to further refine the strategies.

Adnan, Akbar and Wang 2023 define a consensus method as one in which outcomes are determined by the collective agreement of the group. They further reiterated that consensus in Delphi studies is typically achieved when at least 70% of participants rate an item as 3 or higher on a Likert-type scale, with a median score of 3.25 or above. In this study, the researchers calculated the final consensus levels, defined by a threshold of 70% agreement. Trends across the three rounds were examined to demonstrate convergence of expert opinion. Final qualitative insights were also summarised to capture the rationale behind expert judgements. This iterative process ensured that the strategies were thoroughly vetted and refined through collective expert input.

### Trustworthiness

To achieve credibility, the researchers ensured anonymity, continuous iteration, feedback from and to the experts, and aggregation of statistical responses, which is known as member checks. In this study, input from the previous round was shared in each new round so that experts could adjust their assessments in light of the group’s reactions to achieve credibility. To ensure dependability, this article made use of a range of experts who are well-conversant with the subject of inquiry. This lessens prejudice and guarantees a diversity of viewpoints. Confirmability was assessed by maintaining a detailed description of the e-Delphi collection and analysis processes. This covered the number of rounds, the mechanism for providing feedback, and the method for choosing the experts. Transferability was established using verification of the applicability of e-Delphi findings. A summary of the group’s responses was provided while keeping the identities anonymous. This facilitates the process of agreeing while preserving the validity of individual viewpoints.

### Ethical considerations

Ethical approval was obtained from the University of Pretoria, Healthcare Science Research Ethics Committee (Reference number: 497/2022).

## Results

Table 2 presents the results of expert ratings of five strategies across five principles: Validity, Importance, Reliability, Applicability and Clarity. Experts rated each strategy using a 4-point Likert scale, where:

1 = Strongly Agree

2 = Agree to Some Extent

3 = Disagree to Some Extent

4 = Strongly Disagree

**TABLE 2:** Consolidated survey rounds experts’ ratings for a given strategy and principle.

Principles	Validity				Importance				Reliability				Applicability				Clarity			
Ratings	1	2	3	4	1	2	3	4	1	2	3	4	1	2	3	4	1	2	3	4
Strategy 1	37	1	0	0	37	1	0	0	35	2	0	0	38	0	0	0	37	1	0	0
Strategy 2	38	0	0	0	37	1	0	0	36	2	2	0	35	2	1	0	33	4	1	0
Strategy 3	37	1	0	0	38	0	0	0	33	2	0	0	34	3	0	0	34	4	0	0
Strategy 4	30	3	4	1	31	2	4	1	30	6	1	1	30	4	0	4	31	2	1	4
Strategy 5	33	4	1	0	34	3	1	0	33	2	1	2	33	4	0	1	30	3	4	1

**Total**	**175**	**9**	**5**	**1**	**177**	**7**	**5**	**1**	**167**	**14**	**4**	**3**	**170**	**13**	**1**	**5**	**165**	**11**	**6**	**5**

*Source*: Moeti, C., 2024, ‘Development of strategies to incorporate indigenous placental methods for women birthing at healthcare facilities in Tshwane District, in Gauteng Province’, Unpublished thesis, University of Pretoria, South Africa.

Strategy 1 received overwhelmingly positive ratings across all principles. This may indicate a very high consensus and strong endorsement. Validity and importance received the highest consensus. Clarity and applicability show slightly more disagreement, suggesting areas for refinement. Strategy 2 also rated very positively, though moderately less unanimously than strategy 1. Minor disagreement appears in its reliability and clarity, with a few experts selecting ratings of 3 or 4. This still shows strong support overall. For instance, this principle managed to have an overall of 37 ratings marked as strongly agree and 1 as agree to some extent, and no ratings in disagreement. Clarity was consistently rated highly, with unanimous strongly agree responses across all three rounds. Strategy 3, similar to strategy 2, has high agreement and minimal disagreement.

There is slightly more variation in applicability and clarity, but there is no strong disagreement. Strategy 4 shows the most diverse ratings and a lower consensus. For instance, validity: this principle received an overall of 30 ratings of strongly agree, 3 of agree to some extent, 4 of disagree to some extent, and 1 of strongly disagree. Clarity was notably contested, with 4 ratings of strongly disagree. This suggests this strategy may need revision or clarification. Strategy 5 reflected moderate consensus, accompanied by some disagreement. For clarity, the principle received an overall of 30 ratings of strongly agree, 3 of agree to some extent, 4 of disagree to some extent, and 1 of strongly disagree. This indicates mixed perceptions, possibly because of ambiguity or relevance concerns.

This study employed the Average Per cent of Majority Opinions (APMO) Cut-off Rate, which involves calculating the sum of majority agreements and disagreements, multiplying by 100, and dividing by the total number of opinions expressed (Adnan et al. 2023). The APMO formula is as follows:

$$\text{APMO}=\frac{(\text{Majority Agreements})+(\text{Majority Disagreements})\text{ X }100}{\text{Total Opinions Expressed}}$$

Table 3 illustrates a breakdown of the percentage of agreement for each principle based on the consolidated responses from 38 experts across five strategies. The results indicate that all five principles show very high levels of agreement, with percentages ranging from 94.1% to 96.8%. Clarity has the lowest agreement rate, suggesting it may need further refinement or clarification. The consistency across principles indicates strong consensus among the experts, validating the robustness of the strategies evaluated.

**TABLE 3:** Breakdown of the percentage of agreement.

Principle	Strongly agree	Agree to some extent	Total agreement	Total ratings	% Agreement
Validity	175	9	184	190	96.8
Importance	177	7	184	190	96.8
Reliability	167	14	181	186	96.3
Applicability	170	13	183	189	96.8
Clarity	165	11	176	187	94.1

*Source*: Moeti, C., 2024, ‘Development of strategies to incorporate indigenous placental methods for women birthing at healthcare facilities in Tshwane District, in Gauteng Province’, Unpublished thesis, University of Pretoria, South Africa.

The refined and validated strategies to incorporate indigenous placenta disposal for women birthing in the Tshwane district are illustrated in Table 4.

**TABLE 4:** Refined and validated strategies to incorporate indigenous placenta disposal methods.

Theme	Goal	Objective	Strategy	Rationale
Knowledge of indigenous placenta disposal methods, including placenta burial	To promote an understanding of indigenous placenta disposal methods to empower stakeholders with the knowledge to enhance an objective approach in managing women opting to dispose of their placentas through the indigenous placenta methods.	To recognise various indigenous placenta disposal methods practised in Tshwane District.	Foster collaboration between midwives and indigenous groups.	The strategy will equip midwives to understand the cultural context and specific health needs of their communities, enabling them to offer guidance where necessary.
		To prevent and control infections from the placenta during its transportation for its disposal.	Apply infection prevention and control measures when handling, transporting, storing and disposing of the placenta indigenously.	
Cultural respect	To preserve, promote, and sustain cultural respect for the various indigenous placenta disposal methods.	To promote non-judgemental and respectful maternal healthcare to indigenous pregnant women and their families.	Provide education and awareness on maternal cultural care.	The strategy will enhance trust and communication between midwives and indigenous women.
			Provide culture competency training for midwives.	
Recognition and acknowledgement of existing legislation	To ensure the availability of clear protocols in the healthcare facilities that guide midwives on the management of placenta release for indigenous disposal.	To enhance the standardisation of placenta release for indigenous disposal and safety precautions.	Develop policies and guidelines, recognising indigenous customs.	The strategy will ensure that indigenous placenta disposal practices are respected and integrated into modern healthcare settings, providing culturally sensitive care for indigenous women.

*Source*: Rasweswe, M.M., Peu, M.D. & Mulaudzi, F.M., 2021, ‘The Indigenous perspective of the meaning and treatment modalities of dysmenorrhea among the Batlokwa women of Limpopo Province’, Unpublished thesis, University of Pretoria, South Africa.

### Strengthening partnerships

#### Foster collaboration between midwives and indigenous groups

The strategy may encourage ongoing dialogue, decision sharing and partnership between healthcare providers and indigenous communities to ensure culturally appropriate care and mutual understanding.

### Safe and culturally sensitive practices

#### Applying infection prevention and control measures

The application of this strategy may ensure that all placenta handling, transport, storage and disposal practices meet clinical safety standards while accommodating indigenous customs.

### Cultural awareness in maternal care

#### Provide education and awareness on maternal cultural care

The strategy may equip midwives with knowledge about indigenous beliefs and practices surrounding childbirth and placenta care, which may enhance empathy and responsiveness.

### Building cultural competence

#### Provide cultural competency training for midwives

The strategy may assist in the delivery of structured training programmes that build midwives’ skills in delivering culturally safe and respectful care to indigenous women. It may also facilitate the development of skills and attitudes that promote effective maternal care for indigenous women, leading to positive outcomes.

### Policy integration of indigenous customs

#### Develop policies and guidelines, recognising indigenous customs

This strategy may be used to establish the formal hospital protocols that acknowledge and support indigenous placenta disposal traditions, ensuring consistency and institutional support.

## Discussion

The draft strategies were collaboratively formulated by a panel of experts to uphold indigenous cultural values while ensuring compatibility with established clinical protocols. These strategies are unique because they are culturally grounded, co-created with indigenous and clinical stakeholders, and designed to bridge traditional placenta practices with hospital protocols. They add value by promoting cultural safety, improving patient experiences, strengthening trust between communities and healthcare systems, and guiding policy development for more inclusive maternal care. The developed strategies will foster collaboration between midwives and indigenous groups. This is supported by a study conducted in Canada by Benoit et al. (2024), where it was found that indigenous midwives played a crucial role in advocacy on the primary care team, helping indigenous youth develop the skills to go through healthcare systems that are often discriminatory and oppressive towards them.

According to WHO (2007), Standard Precautions for the Prevention and Control of Infection aims to minimise the spread of bloodborne and other infectious pathogens, protecting against transmission from both known and unknown sources. Improper placenta disposal may carry risks of infection, especially in areas with poor sanitation (Nnagbo et al. 2023). The developed strategies are therefore crucial in curbing the spread of infection through indigenous placenta disposal. They will ensure that indigenous women are provided with information on how to handle, store and discard the placentas at home in adherence to their cultures when discharged from the healthcare facility.

The WHO (2003) recommended supporting ‘culturally appropriate’ maternity care services to improve maternal and newborn health. Implementing the strategies that provide maternal culture care awareness and culture competency training to midwives will enhance the relationship between the midwives and the indigenous women, leading to improved health outcomes. This has been proven by the study findings of Jones, Lattof and Coast (2017) from countries such as Australia (targeting the Indigenous Aboriginal and Torres islander women); the United States mentioned that the inclusion of trained midwives on various cultural practices enhances the culturally appropriate maternal healthcare.

The unavailability or limited protocols in the healthcare facilities guiding midwives on safe release of indigenous placenta not only frustrates the midwives but also exposes the indigenous women and their families to health hazards (Moeti et al. 2025). These strategies may guide policymakers in developing guidelines or policies relating to indigenous placenta disposal. Health Ministries in countries such as Australia and Canada have developed policies that govern and guide the culturally safe transportation and disposal of placentas in the healthcare facilities (Cohen 2021).

## Conclusion

The refined and validated strategies aim to foster cultural respect and sensitivity, which can build trust between the midwives and indigenous women. Implementing these strategies in hospitals involves integrating culturally respectful guidelines into policy, training staff in indigenous practices, providing logistical support for placenta handling, engaging with indigenous communities, documenting patient preferences and continuously evaluating the process to ensure respectful and practical care. These strategies in Tshwane district healthcare services may ensure a safe indigenous placenta disposal, minimising the risks of infections to humans, animals and the environment. The strategies may also assist in the provision of a holistic health approach, leading to improved healthcare. Further research is strongly recommended to develop comprehensive guidelines and protocols for indigenous placenta disposal in hospital settings. While initial strategies may offer culturally respectful approaches, additional research can ensure broader cultural representation across diverse indigenous communities, recognising that placenta practices vary significantly.

The strategies may ensure the sustenance of indigenous practices, which will benefit community leaders and elders. The strategies are not limited to covering Tshwane district healthcare facilities only; their implementation will benefit any healthcare facilities globally.

These strategies may be reviewed after a 3-year period unless new evidence comes forth, changing the recommendations in some areas of these strategies.
